# The Introduction of Simulation Sessions to the Psychiatry Teaching Programme for Graduate Entry Medical Students at Swansea University

**DOI:** 10.1192/bjo.2025.10283

**Published:** 2025-06-20

**Authors:** Rhiannon Kihara, Julia Kramer, Angharad Piette, Alistair Carley-Smith, Ruth Wolfe

**Affiliations:** Swansea Bay University Health Board, Swansea, United Kingdom

## Abstract

**Aims:** Simulation is an established part of medical education, but has taken longer to become embedded within psychiatry. Our aim was to introduce Simulation to the teaching programme during medical students’ speciality attachment, using a range of stations to provide exposure to specific mental disorders for all students. Identified learning objectives were to practice psychiatric history taking and mental state examinations, to summarise and present this information and to generate differential diagnoses and propose management plans.

**Methods:** The teaching programme was adjusted to include half a day of Simulation stations during students’ first week, after they receive teaching on history taking, mental state examination and risk assessment during induction.

The clinical tutor developed an introductory presentation, outlining learning objectives and expectations. Each cohort of 14 or 15 students were divided into four small groups and each group instructed to rotate around four different stations, providing exposure to different and realistic scenarios. Clinicians receive stations in advance, allowing time for preparation and familiarisation with the scenario.

We liaised with Simulation leads at two local hospitals for advice about running the stations and debriefing methods. Debriefing is provided during each station, and as a group at the end, and a template has been developed, ensuring the process is in line with Health Education and Improvement Wales and the Association for Simulated Practice in Healthcare expectations.

A Simulation room has been developed at the Education Centre. Simulation teaching is provided for each student cohort.

**Results:** The Simulation stations were introduced in September 2024, and have been carried out for four student cohorts to date. Feedback has been highly encouraging, with all medical students (total 46) rating the sessions as 4 or 5/5 in terms of how useful they have been.

**Conclusion:** Allowing medical students to practice history taking, mental state examinations, risk assessments and capacity assessments has clear advantages. Students can make mistakes, without exposing patients to avoidable harm, debriefing leads to deeper understanding of how diagnoses are made and treatment plans formed. Individuals may identify gaps in their existing knowledge or areas of communication skills which they wish to develop. There is scope to develop this teaching further. We are working with simulation leads at local hospitals, developing VAR headset modules to simulate patients with psychosis, mania, delirium. New stations are being written, to cover situations arising in different clinical settings, demonstrating how relevant comprehensive psychiatric assessment will be in all areas of medicine.

